# Encouraging Undergraduate Marketing Students to Reflect on Critical Thinking and the Digital Gender Divide

**DOI:** 10.3390/ejihpe11030069

**Published:** 2021-08-19

**Authors:** Elena González-Gascón, María D. De-Juan-Vigaray

**Affiliations:** 1 Departamento de Estudios Económicos y Financieros, Facultad Ciencias Sociales y Jurídicas de Elche, Campus de Elche, University Miguel Hernández, 03202 Elche, Spain; elena.gonzalez@umh.es; 2 Department of Marketing, Faculty of Economics, University of Alicante, 03080 Alicante, Spain

**Keywords:** digital gender divide, critical thinking, active learning methodology, collaborative work, student’s reflections, quantitative analysis, qualitative analysis, peer evaluation, technological resources

## Abstract

In today’s society where there is an abundance of accessible, complex, and often false information, critical thinking (CT) is an essential skill so that citizens in general and students in particular can make complex decisions based on scientific evidence, rather than on prejudices, biases, and pre-established beliefs. In this context, the purpose of this study is to discover whether Active Learning (AL) methodology, using different technologies, contributes to improving the CT of the student body, applying it to the Digital Gender Divide (DGD). Three questionnaires were used to collect information, using both a quantitative and a qualitative approach. Open-ended questions are included for fuller answers, which are complemented by content analysis of the recordings and virtual presentations made. The results show that the AL methodology favours the development of CT in the DGD in a remarkable way. Likewise, the various technologies implemented in the methodology (e.g., the Google Applications Site, online round table discussions, role-plays, virtual presentations, and forms) are relevant to improving CT in DGD. It concludes by recommending the implementation of AL with CT as in the one carried out, to help prepare better professionals and raise awareness of how to reduce the DGD.

## 1. Introduction

In today’s society, the proliferation of non-objective sources of information and of fake news makes it essential that the public acquire and develop critical thinking skills (CT) allowing them to distinguish lies from the truth, free of prejudice, bias, and pre-established beliefs [1]. In this way, complex decisions may be made based on knowledge of the scientific evidence [2]. This should be one of aims of Social Sciences education [3] and, due to the importance of CT, many universities and institutions of higher education consider it one of the skills acquired as a result of students’ learning process [4].

The aim of this research is to show an educational experience in which, through the active learning methodology (AL), it is intended to increase the student’s critical thinking skills (CT) regarding the digital gender divide (DGD) employing the technological resources in the critical education of graduate students: The future citizenship.

The importance of this study becomes particularly relevant in the context of study. Specifically, these are students in the final semester of the final year of a degree in Business Administration, who are about to become professionals entering the labour market and who, however, confirmed before undertaking the activity that they do not know what the DGD is, and that, except for one student, have not applied CT in their academic studies before. Highlighting the need to develop CT in university students is an unresolved topic in marketing, which must be covered in the shortest possible time, and this educational experience aims to contribute to this.

### State of the Art

Firstly, after reviewing the literature related to this theme [5,6], AL seemed appropriate since previous studies had been successfully shown to be an adequate technique to increase CT. As a result, using AL, students self-reported improving engagement as well as a deeper understanding of course materials. Furthermore, the author of [7] was also inspiring to us for using AL methodology since he reported that the mix of traditional lecturing combined with AL (what we wanted to apply) was a methodology to maintain students’ interest in and attention to specific topics.

Secondly, after knowing that AL had already been proved to be a reliable technique, the literature showed (e.g., [8,9]) that it had been successfully applied in other fields when using debates.

Other AL approaches such as using wikis seemed less adequate [10]. More precisely, these authors indicated that when using wikis, students became anxious and unwilling to participate in the collaborative knowledge-building process. Moreover, as [11] reported, with the use of wikis, students showed their reluctance to share and collaborate, impeding them from contributing to and gaining CT. These facts doubly convinced us of the use of AL and debates in the unresearched marketing field since they seemed both adequate and reliable as they had been tested previously in other fields.

Thus, with the AL methodology, the student acquires responsibility for his/her own learning, considering that knowledge is generated from interaction with other agents and based on reflection [12]. In this environment, the teacher assumes the role of a facilitator, with the task of designing and structuring the different teaching activities with the aim of creating a direct relationship between theory and practice [12], thus achieving the application of theoretical knowledge to the different contexts and situations of his/her life, facilitating the development of CT.

Today’s students not only require the cognitive aspects, but also critical thinking, communication, creativity, and collaboration [13]. For students to become involved in the teaching–learning process, they must be able to abandon the more or less passive role of “just listening” and develop a set of skills:

1. Performing tasks that require thought processes of some complexity.
2. Their active participation in learning.
3. The questioning of one’s own beliefs and values [14].

This includes carrying out a thinking process consisting of the following stages:

1. Planning.
2. Contextualization.
3. Individual reflection.
4. Action/Practice.
5. Collective reflection.
6. Continuous evaluation and improvement [15].

This thinking process has a bearing on improving CT for everybody involved in the process.

Given that there is no single definition of CT applicable to all disciplines at all levels [16], this is defined as the reflective thinking involved in evaluating the important evidence regarding a topic to draw solid conclusions based on that evidence [17]. CT is a highly complex cognitive process, in which reason and thoughtful act predominate, its purpose being to recognize what is just and true [18,19]. Some authors consider CT to be “the art of analysing and evaluating thought with the goal of improving it” [20].

It is a thought process oriented by doing, to action, and is mainly put into practice when problems must be solved [21]. Three generic capabilities are needed for its development: Focus, inference, and judgement; and eighteen specific capabilities [19] among which the following stand out:

1. Judging the credibility of a source.
2. The use of existing knowledge (prior knowledge, including Internet material, knowledge of the situation, and previously established conclusions).
3. Making value judgments.
4. Inducing and judging inductions and arguments, including the arguments and inferences of the best explanation.
5. Handling errors appropriately.
6. The use of appropriate rhetorical strategies for discussion and presentation, whether oral or written [19].

An extensive review of the different capabilities included in CT can be found in [16].

On the other hand, when practicing rhetoric, the student must keep an open mind, also considering those ideas that may initially seem unattractive. In this process, listening to the opinions of other colleagues is one more piece of CT and it is necessary to understand them, know who they are, how they think, what they need, and what they think they need [22]. In this sense, conducting activities in a university environment that favours students’ CT improves the quality of their ideas on current issues, since they review concepts that had not been questioned on an academic and intellectual basis [23], one of vital importance today being the DGD.

Successful developments in the Information and Knowledge Society (IKS) require graduates to have professional digital skills, with the ability to adapt to new social demands [24]. Precisely, the gap between men and women in terms of technology is known as the DGD, which has been the subject of recurrent study in recent decades [25].

When introducing sensitive topics into course content, such as in this case with DGD, debates [26], roundtable discussions [27], and role-playing [28] can be very beneficial since they are a good tool to improve information gain.

To prepare for good CT learning, a multitask AL activity is proposed, which is in line with previous studies [29,30] that used, among other tools, teamwork, oral presentation skills, and reasoning abilities. In this study, these instruments are extended and include new ones, which previous researchers have used and proved their adequacy [27,31,32]. However, each instrument was employed singly in a specific study and the challenge in this study is to use a variety of them as a preparation for CT. More precisely, the tools included are the following: Interacting with guest speakers, used earlier by [31,33,34], watching documentaries [31], roundtable discussions [27], and role-playing [28]. In summary, the spectrum of pre-activities used in this study is part of its strength because they were all used as part of the same course to prepare the same students for CT, and no previous literature was found with such a wide range of techniques used at the same time.

Moreover, the literature states that there are differences in self-perception manifested by men and women in the management of information and communication technologies (ICT), with self-perception being greater in men than in women [35,36]. However, other studies suggest that the DGD is narrowing [37]. For more information, consult the work of Ortega and Gómez-Trigueros [24].

These two divergent approaches to the DGD highlight the need to go further and to make both male and female adults, experienced at a university education level, digital natives, critically reflect. The research questions that arise are as follows:

- RQ1. Is it possible through AL to help to improve students’ CT in the study of the DGD?
- RQ2. Do the students consider that communication skills are relevant to improving their CT?
- RQ3. Which technologies best favour the development of CT in learning about the digital gender gap?

The results confirm that the CT has helped to improve the DGD in the classroom. In turn, the communication skills and various technologies implemented in the classroom through AL have been relevant to improving CT in the subject.

This article is organised as follows. Firstly, the Materials and Methods section sets out the participants, the equipment and the task material used, the evaluation questionnaires employed, and the method design applied. Secondly, the results part deals with the results of the previous activities pursued in order to improve CT and those of the questionnaire used to collect students’ knowledge of the methodology used in pre-practice tasks on DGD and CT, the results concerning the preparatory phase, the qualitative results from the AL Session on CT and the DGD, and the results of the peer review questionnaire and those of the final debate. Next, the results on the use of technologies and communication skills in CT learning on the DGD, and the quantitative and qualitative results on acquired skills are presented. Finally, the conclusion and discussion parts bring the study to a close.

## 2. Materials and Methods

### 2.1. Participants

The study was carried out with a theoretical and convenience sampling [38,39] with 35 participants, divided into two groups, studying Commercial Distribution and Retailing (Marketing discipline) as part of the 4th year of a degree in Business Administration and Management at the University of Alicante (Spain). The first group comprised 20 Spanish students (7 women and 13 men) taught in Spanish (hereinafter referred to as SP) and the other group comprised 15 international students from various countries: France (5), Belgium (4), Belarus (1), China (1), Germany (1), Poland (1), Ukraine (1), and the USA/Mexico (1); being 6 women and 9 men, taught in English (hereinafter referred to as INT). It was verified that students were completely unaware of the DGD theme and that only one student knew what CT was. As in other cases, the students participated in the study and the anonymous questionnaires linked to it as part of the subject activities, having as an incentive to do so the mark associated with the activity. Before conducting the study, participants voluntarily signed to give their consent to the use of their answers and photographs.

As [39,40] state, in qualitative studies, the decisions concerning the sampling methods reflect the researcher’s premises according to what makes a credible, reliable, and valid sample to approach the problem statement. The correct sampling is of crucial importance in the research [41] and the literature shows that small samples are valid in qualitative studies [27,28,33,34,42,43].

In this case, 7 pre-activity preparatory sessions on CT and DGD for 35 students (7 groups, 4 Spanish and 3 English), the analyses of 3 questionnaires, PowerPoint presentations, and their recorded video analysis, combining quantitative and qualitative techniques, were conducted. Category saturation to give answers to the research questions was reached with this number of participants and activities [44] and the sample was considered correct for the purpose and type of study [38].

### 2.2. Equipment and Task Material

Computers with Internet access were necessary for this task and students had to have access to Zoom and the Google Site (document, spreadsheet, and presentation software accessible online, and forms) in order to use them online during the group sessions and discussions, as well as to resolve the activity simultaneously, and mobile phones provided with message apps. Teams were given a topic (see Table 1) on the DGD and the criteria by which to evaluate it as part of their critical appraisal coursework [45]. In accordance with Barron [46] and Blatchford et al. [47], the students had support materials to ask themselves the appropriate materials and to help them identify relevant and irrelevant evidence. In this manner, an Excel spreadsheet was prepared for each group with the following instructions for the DGD topic:

1. Undertake a simultaneous search using key words and state the search sequence in the cell designated for it.
2. Each team can propose as many search sequences as they wish, the minimum number being two.
3. State the search engine used for the search.
4. Enter the data obtained: Link, source, date, and the importance of the information found (where 1 means “I don’t think it is important for the development of my critical thinking” and 10 means “I think it is very important, very relevant, for the development of my critical thinking”).
5. Compare the first five results that appeared in that search and annotate in the match table those that match and those that do not.
6. Indicate observations.

### 2.3. Evaluation Questionnaires

Three questionnaires were used to evaluate the task and support material in terms of helping students to complete their critical appraisal. Q1 and Q3 used in this study were developed ad-hoc by the authors (see Table 1). Both collect quantitative and qualitative information. In the case of quantitative items, both used Likert scales where 1 is “nothing at all” and 5 is “totally”, as well as numerical scales (from 0 to 10) to assess the extent to which the different activities develop the skills and CT. In this way, it was intended to check whether participants have similar beliefs about the subject matter of study, with the answers offered being richer in nuance [48].

There was also an evaluation of group discussion as regards their contribution to the critical appraisal assignment.

### 2.4. Method Design

The class of Spanish students was organized into 4 teams of 5 people each (SP1, SP2, SP3, SP4) and the class of international students into 3 teams, again with 5 people in each (INT1, INT2, INT3). The methodology used based on [12,15] includes six stages, as seen above. These stages have already been followed by the authors in other educational experiences [50], but in this AL proposal in particular, the sequential structure proposed by these authors has been slightly altered by the very nature of the activity and the experience gained, with both individual and collective reflection becoming of key importance. In this format, two individual reflections, instead of one, at two different moments in time, that is before and after the presentations, are made in Session 3. This allows the evaluation of learning not only in DGD, but also in CT. The different phases fit into four distinct sessions, as detailed in Table 2 and explained below, taking into account that the length of each session is 1 h 50 min. Moreover, this AL application has a series of pre-activity preparatory sessions on Critical Thinking and the Gender Digital Divide with ITCs, which take place over the previous 10 weeks.

#### 2.4.1. Pre-Activity Preparatory Sessions on Critical Thinking and the Digital Gender Divide with ITCs

From the beginning of the course, teams are formed to promote collaborative work [51], and through ten previous practical sessions, the students receive different training and guidance to improve their skills in technological resources and communication and argumentative skills in Critical Thinking in Commercial Distribution and Retailing. Specifically, the activities that arise cover the content objectives of the subject syllabus [52], methodologically focused on:

1. Learning to search for news from reliable sources, filtered by dates, and then relate the real world to the subject matter, all with a high, complex difficulty [53].
2. Checking sources; making recommendations to companies in real situations [53].
3. Participation in role-plays [28,42,54].
4. Preparing and participating in roundtable discussions [27,43].
5. Watching documentaries and analysing information to draw conclusions and design lines of research [32].
6. Interaction with a guest speaker from a successful real retail company [31,33,34].
7. Analysing the ethics of buying and selling products in retail through a discussion [55].

#### 2.4.2. Active Learning Session on Critical Thinking and the Digital Gender Divide

Session 1 (Introductory).

This first session explains, on the one hand, the objective of the activity, which is to develop critical thinking with regard to the ethical principles in retailing, considering the gender perspective and, more precisely, the gender digital divide; and, on the other hand, to develop online communication skills. The materials to be used by students are also explained (see Section 2.2), as well as the methodology to follow. Students are then offered possible DGD topics, with the option to work as part of a team or to do so individually. In both groups, the students choose to work as a team and decide on their subjects without overlaps (see Table 3) [56]. This decision makes it possible to verify that the methodology used in previous activities (Section 2.4.1) has given a positive result, facilitating a good collaborative working environment [51].

It is then set out what Critical Thinking is, and the explanation includes a video on the subject, as well as the recommendation that they view an interesting TED talk on it.

As Barron [46] and Blatchford et al. [47] suggest, it is important to teach students standards of social interaction and to understand that the function of their arguments is to persuade listeners of the validity of them. In this way, according to the authors, the next task is to explain the rules of social interaction to be used and to define clear and specific approaches for each SP and INT team. Specifically, two topics (A, B) were assigned to SP and three (A, B, C) to INT (see Table 3). The SP groups’ presentations comprised two “duels” where each pair of teams had to make the best possible argument for their, up to this moment, unknown subject against their opponent. All of this using CT, a methodology the had not used before, in order to convince the teacher and the other two teams. These teams in turn evaluated them according to the rubric of Questionnaire 2. The INT groups presentations were not competitive, with the premise that they should make their arguments in relation to their also absolutely unknown subjects as persuasive as possible [57]. All the teams were aware of being evaluated by the other teams as well as the teacher.

Individual reflection (Preparation week).

After Session 1, the seven teams had one week to prepare for Session 2, at the beginning of which the Q1 Questionnaire on methodology and learning from the previous activities was completed. They had to start by preparing a team presentation, including a PowerPoint, on their topic to be presented in Session 3, based on the Excel material they had been working on (see Section 2.2) producing a team Excel file showing the search procedure with their observations. It was noted that during that week there was a local public holiday and, therefore, one of the days of that week was a holiday for the students.

Session 2 (Online Workshop).

In this session, using the ZOOM platform, students were organized into breakout rooms. The teacher tutored each of the groups while the rest worked in parallel, on the task. This session clarified any queries that may have arisen during the preparation week and provided more precise guidelines on Session 3 (e.g., all the members had to present; presentations would last between 10–15 min per group; the hypothetical situation for the session was: “Each group will imagine that it is in a job interview and must convince the interviewer of their arguments. The best presentation will be awarded the job. Any one person could be invited to ask questions and the participants will be ready to answer”). In addition, at this time, the Q2 Questionnaire was handed out with the Critical Thinking Assessment rubric [49] so that students knew the scale on which they would be assessed.

Session 3 (Presentations on Critical Thinking and the Gender Critical Divide).

With the consent of the students, this session was recorded for later viewing by the groups and the teacher. Students set out their arguments, as planned. The presentations lasted approximately 15 to 20 min. It was not necessary to draw attention of any group to the duration of the presentations, although in the preparatory sessions, this was necessary since they did not respect the time set.

Session 4 (Debate and Feedback Session).

After one week, which allowed teams to view all the presentations, the feedback session was held in which students answered the Q3 Questionnaire. In this session, the debate, in which students exchanged their views on the other teams’ presentations and the DGD, takes place. The debate was very intense and active compared to the debate that, for example, took place in the third week of the course. All the teams in both groups, SP/INT, participated and produced certain discrepancies of opinion as to the degree of agreement and disagreement with the issues presented.

## 3. Results

Below are the results from the different questionnaires, in the order of the sessions and the stages corresponding to Chirino et al. [15] and Huber [12]. The different sections set out both the necessary quantitative and qualitative results to enhance and nuance them [48].

### 3.1. Results of the Previous Activities. Questionnaire Q1

These results correspond to the *individual reflection* stage, which was undertaken on the different methodologies and the learning carried out in previous activities. The different activities (A) carried out throughout the subject to develop CT are as follows:

- A1, round-table discussion undertaken online.
- A2, role play.
- A3, difficult information search and recording a presentation.
- A4, comparing current information sources and making recommendations in real-world situations.
- A5, watching documentaries and subsequent reflection.

Table 4 shows the percentage of students having no previous experience of the different activities and methodologies. As noted, there are important differences between the Spanish student group and that of the international students. While the SP group had almost no previous experience in activities such as round-table discussions or recording presentations, the INT group states, in particular the women, having done so before. The activity in which they had the most previous experience was watching documentaries and later reflecting on them. In general, it can be concluded that most of them had not experienced similar activities.

### 3.2. Results of the Preparatory Phase (Material for Session 2)

These results belong to *the contextualization stage*, in which the teacher tutors and assists in contextualization for each of the teams. In relation to the results obtained for both groups, they are practically the same. Students warned us that by entering the subject-related keywords, they all got practically the same outputs (e.g., Table 5, SP1 as an example), slightly altering the order of appearance of the sources, if possible. That is why they decided to repeat the search with other keywords on topics they did know about, and they detected that the results were according to their previous Internet use based on cookies (e.g., Table 5, SP3). Finally, they decided to look for reliable secondary information that could help them support their argument (e.g., INT3). The students acknowledged that, in the activities of previous weeks, the searches had not been as scrupulous as per this time and they realized critical thinking was required.

### 3.3. Qualitative Results from the Active Learning Session on Critical Thinking and the Gender Digital Divide (Session 3)

In the *practical action stage,* the students gave their presentations. Once given, the PowerPoint presentations were subjected to a computer and section content analysis (SP/INT). The technique used to select the answers given by the students, as evidence of the achievement of the objectives set, is their importance and relevance [58].

A double vertical analysis was undertaken. On the one hand, the presentations were analysed individually from the beginning to the end, without linking them to each other, identifying the topics. On the other hand, the recordings of Session 3 were reviewed, complementing the PowerPoint slides presented. In the second stage, a horizontal analysis was performed, linking all the presentations to identify the common themes that, in effect, the students identified, to see to what extent they have done so. Thus, in each PowerPoint, the content was reviewed, and the text related to the topics of CT and the DGD was marked. Expressions and comments important to the AL approach were identified, the sources used for drawing it up were taken into account, and conceptually put together using the constant comparison method with colour cards [58]. The words that the students used on a recurring basis (common to most and all that were frequently mentioned) were identified.

Session 3 results show that students learned how to work through critical thinking and the digital gender divide. The conclusions of the teams, as expected, were different depending on how thorough the critical thinking process had been carried out.

Table 6 shows the results in which can be seen how all the teams, to a greater or lesser extent, have assimilated CT and are aware of what it is and how they have applied it. As for the DGD, the answers are more diverse. In general, the teams detected that there is a digital gender gap, to a greater or lesser extent, and only one team (INT1), which did not provide sources for its arguments, indicating that it did not have time to carry out the tasks (it must be remembered that there was a public holiday in the preparation week and the international students stated in their comments that they could not work due to “the holidays”), pointed out that the DGD no longer exists. However, the rest of the SP and INT teams were able to present a coherent, orderly, and justified discourse (see Table 6).

As for ICTS, the opinions of all the teams both SP and INT (except INT1) detected their importance today.

### 3.4. Results of the Q2 Peer Review Questionnaire and the Final Debate (Session 4)

This block of results corresponds on the one hand to the individual reflection stage, in which students must, using the rubric of the Q2 Questionnaire, evaluate their peers. As shown in Table 7, in relation to peer evaluation, the average grade of the SP group students is 7.2, while it is 7.9 in the INT group, whereas the teachers awarded a mark of 8.5 to the SP group and 8.1 to the INT group. There was a greater dedication to the activity among the SP students’ teams, with an average of between 8 and 10 h spent individually in three of the teams compared to 1 h in the fourth team. They point to an average of about 2 h of group work. The three INT teams reported that they spent an average of between 2 and 3 h per member, and about 2 h on average in a group, on the project.

On the other hand, Session 4 corresponds to the stage of collective reflection, which is carried out on the subject using an informal debate format, the different methodologies used in the undertaking of the activity, and the learning carried out in the previous activities.

In this session, the teacher informed the SP and INT students of the marks obtained through the peer-evaluation method. They were displayed anonymously, indicating the average of each group and the winning team.

Finally, delegates from each team ended Session 4 by giving the teacher feedback on the entire AL.

### 3.5. Results on the Use of Technologies and Communication Skills in CT Learning on the Digital Gender Divide. Questionnaire Q3

Below are the results corresponding to the *evaluation and continuous improvement stage.* The results corresponding to both quantitative and qualitative items are presented separately.

#### 3.5.1. Quantitative Results on the Activities Carried out

Table 8 shows the averages (with their typical errors) of the five activities proposed to increase CT. They have been measured using a 5-point Likert scale. All activities score positively above the arithmetic mean (3). The highest valued are the online round-table discussion (A1), the search for information to generate a presentation with recorded voice (A3), and collating sources of information to make recommendations in real situations (A4). The activity that obtained the lowest score was viewing documentaries and drawing conclusions and reflecting on them (A6). It is worth mentioning that there is a negative correlation between having previously carried out the activities and their assessment to promote CT.

#### 3.5.2. Qualitative Results on the Activities Carried out on CT in the DGD

To the question “After doing the activity, do you think that the gender digital divide can affect the retail world?” 100% of INT students said “yes”. The different nuances obtained in the answers allowed the enhancement of the knowledge. As an example, some answers are shown:

*“This is actually a true fact. Could affect the ways they show the products through advertising, they could be biased. This could be a huge issue if people are aware, and companies may face some problems and be infamous for it”*

(INT).

*“As we talk about in our presentation, it can affect several aspects, such as marketing for example, where we absolutely need internet and to know how to use it to work. If people don’t have internet or just don’t know how to use it, it will be a big problem for them. Then, same for hiring people, we need to register online to an offer.”*

(INT).

Among the SP students, the percentage that agreed with this question was 95%. We have highlighted in the first instance the comment expressing disagreement:

*“Everything depends on where we live, and the definition used, if we focus on Spain and use the INE (Spanish National Institute for Statistics) definition, we see that, in general, the gender gap is minimal.”*

(SP).

This is a clear example of how qualitative responses permit showing nuances as opposed to quantitative ones. Thus, the answer “no” is nuanced by the environment in which “Spain” is located. In any case, it denotes a high level of CT on a previously unknown topic. Other positive responses may be found below:

*“The digital divide is something that can really affect retail, since sales and sales differentiated by gender can be affected if everyone does not have the same opportunities to use online commerce or cannot discover the wide variety of offers which commerce in general makes available.”*

(SP).

*“Because as we saw in the activity, CVs are classified according to the words used and this can mean that women do not have the same opportunities to work in retail as men.”*

(SP).

The next question posed “After doing the activity, do you think that the society that is presented in the media, on the Internet and in advertising related to the gender digital divide is real?” showed greater discrepancies, between and within both the INT and SP groups. Thus, among the SP students, 40% think that it is definitely real, and the media show it. However, a 60% majority opposes this position. In the INT group, there is an equal split at 50%. Below are some explanations, both for and against.

*“It is implied that this gap is “reducing”, but it really is something that still exists, although it is true that it is to a lesser extent, but it still exists. Therefore, in sectors such as toys we are presented with obsolete ads where they associate gender roles with certain activities and that should not be. It should be all unisex.”*

(SP (60%)—it is not real-, the DGD exists, although the media do not show it).

*“I don’t think it’s real, we always see a “standard” society, which is far from reality, in communication, advertising and marketing campaigns. We always see a very similar profile, with a good image, a good presence, from the upper-middle class, etc. The best example is the perfumery sector, always top society, which if you look at the clientele is obviously not so. Therefore, I believe that it is not real.”*

(SP (40%)—it is real- and the mass media are biased. The gap exists, but the media does not show it).

In both comments from the SP group, it is verified that although they have answered the quantitative item differently (a yes and a no), both consider that the DGD exists, but one perceives that the mass media hide it. The detail of these responses allows us to see the importance of complementing quantitative data with qualitative data.

*“Because there are lots of instances where women’s problems have been minimised and swept under the carpet to satisfy the needs of men. Definitely in the media, there needs to be a change: why is it just “soccer” when men play it, and “women’s soccer” when women play?”*

(INT (50%) which points out that mass media are biased. The gap exists, but the media does not show it).

*“Yes, we are confronted with this problem every day. A study shows that in Asian countries, as the Asian Development Bank has observed, the majority of women-led enterprises, especially in developing countries, are small-sized businesses with low output levels, limited growth potential, thin price margins and very little capacity to bear inventory and customer service overheads. This leaves them at a distinct disadvantage in the platform marketplace with inexorable commission rates and unfriendly terms of service.”*

(INT (50%) Positive, the DGD exists and in the report used, this is shown).

As in the case of the SP groups, both comments in the INT group show that the same thing is true. The students consider that the DGD exists, although one has answered in the affirmative to the question and the other in the negative.

The third and final question shows total agreement in their answers, with 100% affirmative in both INT and SP groups. “Do you think that it is important to have well-developed critical thinking to face up to non-egalitarian information in the media?”:

*“Possessing critical thinking helps you see things more clearly. Even when considering your own opinions. You learn from others and they from you, mutually beneficial. It is also good to know what is “OK” or not in the non-egalitarian information that is offered to us”*

(SP).

*“The truth is that we always have to think critically when analyzing any information, but when it comes to seeing the gender differences, we have to analyze it even more. I think this is because with the gender theme there are many taboos depending on culture. In addition, the data must be viewed and compared with various sources of information that are not adequate”*

(SP).

*“Of course, for me it is very important. You have to have critical thinking at all times, it’s not because the media or the Internet are saying something that it means that it is true. You have to know how to take a step back from the information conveyed, in order to avoid being mistaken or believing in false information”*

(INT).

*“Your web searches are based on your interests and history of searches so it might be false information. I personally use a specific browser that does not save my information, so my articles won’t be matched to my own interests”*

(INT).

#### 3.5.3. Quantitative Results on Acquired Skills

The different skills developed from the educational experience in which, through the active learning methodology (AL), it is intended to increase students’ critical thinking regarding the digital gender gap are as follows:

- Skill 1: Consider different points of view in commercial distribution.
- Skill 2: Information search and analysis.
- Skill 3: Oral communication.

All three are considered important in CT. Table 9 details the means (with their typical errors). The students were asked to evaluate them based on the same range that is used in an examination, i.e., from 0 to 10. In all cases, the skill mark far exceeds the normal pass mark, since they range between good (merit) and excellent (distinction). There are no major differences between the SP and INT groups and, also, neither by gender. The students perceived that the skill that improved to a greater extent was that of the information search and analysis (Skill2), which is directly related to CT. The other two skills received very similar evaluations in terms of importance.

#### 3.5.4. Qualitative Results on the Skills Acquired

The students, in general, improved their communication skills while also being aware of it, as indicated by the SP2 group “*our ability to synthesize when looking for information was improved, since we got a lot of information and we decided to filter it to focus on the relevant bits so that when we presented the oratory ability of each person would be improved*”. As for the manner in which they presented to the teachers and their peers, their attitude underwent an important development. At the beginning of the sessions, only a few had installed the camera. After having carried out the support activities during the previous weeks, in the Session 2 workshop, they entered the class with their cameras on in a totally normal way, for all those for whom the camera worked. Session 3 took place as if it were an actual event, as shown by the instruction: Each group will imagine that it is in a job interview and must convince the interviewer of their arguments. The role-playing games of the previous weeks created the desired effect and many of the teams invented names for their fictitious consulting companies, even going so far as to create logos that they placed in their screen background. All the SP teams and two of the INT ones also looked for background images simulating offices, imagining being in large corporations or expert consultants. Of the seven teams, three of them even decided to attend the session wearing clothing in the same corporate colours as their invented company (e.g., all wearing white shirts), according to the logo of the fictitious companies.

The students did not have any problems when it came to presenting remotely, coordinating very well at all times. They used videos, where necessary, and gave the floor politely and alternatively, as if it were a real consultancy meeting. There were no problematic incidents, and everyone demonstrated a lot of ease using the digital tools necessary to present online.

## 4. Discussion

The participants in the experience will become professionals in just one month after studying the subject and must be ready to enter the labour market as prepared, competent professionals of integrity. However, without exception, before participating in the AL activity, they confirmed that they were completely unaware of what the DGD was and only one student had previously applied CT during her academic career. The result for DGD was very surprising. In fact, previous studies (e.g., [8] (p. 437)) state the following “*In the pre-debate surveys students were asked to provide their opinion on each controversial debate topic question (*Table 3 and Table 4*) as “Yes,” “No,” or “I don’t know” if they were either unfamiliar with the topic or did not have an opinion*”, and the percentage of students indicating that they knew about the topic was 66%. In our study, 97% of the students declared not having heard of DGD, which shows the relevance of pursuing the study on this gender theme.

The results concerning the skills acquired seem to be satisfactory when we compare them with earlier studies. More specifically, Skill #1 (Consider different points of view in commercial distribution) in Table 9 improved within a range of 7.9 to 8.22 (from 0–10), which shows that we are aligned with and even better than previous research [8,9].

In relation to Skill #2 (Information search and analysis) in Table 9, students’ answers range from 8.73 to 9.20 (from 0–10). In fact, before doing the activity, the students stated that they knew how to search. Shockingly, after participating in the AL activities, they admitted that “*they knew how to search, but they recognised that they did not know as much as they thought*”. These results seem to be coherent with [53] in the objective of developing CT.

Skill #3 (Oral communication) in Table 9 shows values between 7.18 and 8.89 (from 0 to 10) indicating improvement in this crucial skill for marketing students. This means that the teams improved their ability to communicate their arguments using the debate format in the DGD context. These results are aligned with [30] in the nursing field.

On their part, in terms of the skills developed by the AL activity, the students perceived that the skill that improved the most was the search for and analysis of information, which, in turn, is directly related to CT [4]. This has been demonstrated in the maturity of many of the conclusions reached, as well as in the quality of the debate held in Session 4.

Students have been able to search, sort, analyse, and synthesize information [20], selecting that which was relevant to them for decision-making related to the proposed topic: The digital gender divide, detecting their thought perspectives (e.g., the DGD exists [24,25], but it is decreasing [37] and they have done so through critical thinking [1]). The students have been able to detect the three stages of the DGD and have solved the mandated activity focusing mainly on the second stage (arrival of the Internet and the beginning of the XXI century), where they have detected a lack of training and knowledge in women for the use of ICTs. All of this was achieved using the CT they learned through the activities carried out during the course.

As for the solutions presented by the students, after looking for and contrasting various sources of information (Session 2) and presenting their arguments (Session 3) on social problems and the world of retail, there are different points of view. In relation to “the gender digital divide and Internet advertising: myth or reality”, the opinions have not been convergent since one of the INT groups concluded that it is a myth that “*The digital gender gap is becoming more and more of a myth*”, although this opinion is not borne out by any sources to support these conclusions, except their own experience of the net. While the rest detected that it is a reality.

With regard to “The gender digital divide; is it smaller in world powers (e.g., USA, China)”, the teams working on this issue are more in agreement in their conclusions. Without doubt, they observed that the DGD is smaller in Europe, but above all, they put the emphasis on what is happening in Asia and in the least developed countries by calling on society to act.

In relation to the topic “has the gender digital divide reduced since COVID-19?”, since only one INT group worked on it, we cannot point out any diversity of opinion or degree of agreement. What is certain is that they warned that the COVID-19 pandemic has accentuated the DGD especially in the most vulnerable countries. They reflected on the fact that “*communities without access to the Internet or with limited connectivity are more isolated and vulnerable and are unable to readily access the public health information and services they need. This will result in deepened social and economic inequalities in the future. A lack of Internet access can also exacerbate an already repressive, harmful and unequal context for women and people of diverse genders and sexualities*”. To summarize, they concluded that the difference between genders is very strong and even proposed solutions for Togo, the Dominican Republic, and Africa, looking beyond Europe.

The authors are really pleased to see how starting from scratch and through the application of CT there is an educational promotion of digital skills and knowledge of equitable access to technology. Likewise, they are also proud to see how the methodological approach used to achieve the objectives through CT [4,10,27,30] has contributed to shining a light on the DGD.

In relation to the questioning of the validity of this study, we agree with those proposed by [8]. Students have mentioned that after AL activities they have changed their minds about a controversial topic [23]. However, it is true that this fact is not automatically a sign of learning. We can say that the AL experience was engaging for the students and that they changed their opinion after finishing it. Furthermore, the objective was to teach them to think critically about an unknown topic, which has been achieved.

Concerning the value gained through public speaking, agreeing with [59], it is true that one team questioned its validity, and higher values were expected for the marks awarded for this activity. Faced with the limitations detected, we assume that, agreeing with [30], forcing them to leave their comfort zone helped them to learn.

Finally, future lines of research could aim to check what consequences the application of CT has in other degrees and at other academic levels, so that the student begins to have unbiased, well-grounded visions from the earliest courses.

## 5. Conclusions

The results allow us to conclude that it is possible, through AL, to contribute to improving students’ CT in the field of the DGD (RQ1) in marketing, that students are aware that communication skills are relevant to improving their CT (RQ2) and that the use of the technologies implemented in the classroom have contributed to their learning and CT regarding the digital gender divide (RQ3).

The AL activity undertaken was carried out in the context of a subject as part of the fourth and final year of the degree course in Business Administration. In the subject, among the skills to be acquired by students at a curricular level, it is required that they be responsible for their learning to develop autonomy and be active in knowledge-building; this develops critical thinking, collaborative attitudes, professional skills, and finally, the ability to self-evaluate.

The final objective of the subject is to sensitize students to the importance of efficient commercial distribution in achieving both the success of the company and the satisfaction of customers. In order to respond to how different methodological approaches can develop social, critical, and creative thinking, educational research, in which the AL methodology with multi-ICTs is used, was proposed.

Specifically, five different activities were used: A1, a round-table discussion undertaken online; A2, a role play; A3, a research activity where students are asked to find difficult information and record a presentation; A4, comparing current information sources and making recommendations in real-world situations; and A5, watching documentaries and subsequent reflection. Results confirm that all the activities have contributed to the development of CT. Therefore, we recommend carrying them out.

In fact, these activities could be implemented together, as we have done, but they could also be combined according to the needs and academic level of the students. However, keeping in mind that the results show that there is a negative correlation between having previously carried out the activities and their assessment to promote CT, teachers could choose which activities are the most appropriate for their students. For instance, the activity that increased CT the most is comparing current information sources and making recommendations in real-world situations (A4). In this sense, we suggest high school teachers design activities in which students can search for information from different sources (minimum three), highlight differences and bias among the results, and, finally, state conclusions.

Another possible application for younger students could be related to watching documentaries and subsequent reflection (A5). At an early age, students are attracted to films (e.g., Disney). Watching documentaries or animated short films in specific topics could therefore awaken their critical spirit by allowing them to see and listen to several different points of view. Afterwards, they could compare and explain the different approaches (e.g., religion, racism, climate change). The idea is to make students think, doing it in a critical way, no matter their age.

The authors also recommend, at any level of the educational system, raising the objectives to be achieved using, whenever possible, CT, as well as designing educational research or AL applications such as the one presented to help prepare better professionals and raise awareness of how to reduce the DGD.

Finally, the SARS-CoV-2 (COVID-19) pandemic has accelerated the use of ICTs for learning in classrooms, forcing teachers and students to use them globally. A return to face-to face learning in classrooms has been planned for the next courses, with all the advantages this entails. It is important to bear in mind all the digital resources that have been used in online teaching so as to continue using them in hybrid or face-to-face situations in future teaching. As John Lennon wrote in his song “Imagine”—“It’s easy if you try”. We recommend that you imagine a world without the DGD, and that you continue to try to achieve it with, among other means, educational experiences such as this.

## Figures and Tables

**Table 1:** Questionnaires used to evaluate the active learning methodology in DGD with CT.

Code	Questionnaire	Objective
Q1	Questionnaire 1: Previous practices methodology and technologies implemented in the classroom	Collect students’ knowledge of the methodology used in pre-practice tasks on DGD and CT.
Q2	Questionnaire 2: Assessment rubric [49]	Teacher evaluation and student peer evaluation.
Q3	Questionnaire 3: Results on the use of communication technologies and skills in learning about CT in the DGD	Evaluate knowledge of DGD and CT derived from undertaking the activity.

**Table 2:** Sessions of the AL Activity on DGD with CT.

Stages of [12,15]	AL Methodology Sessions on DGG Using CT		
*Planning*	SESSION 1	Introductory	Explanation of objectives, materials, and methodology
*Individual* *Reflection*	Implementation of Questionnaire Q1 on methodology and learning from previous activities		
*Contextualization*	SESSION 2	Online Workshop	Online work and teamwork supervised by the teacher
*Practical Action*	SESSION 3	Presentations	Presentations on CT and DGD
*Individual* *Reflection*	Implementation of Questionnaire Q2 peer evaluation		
*Collective* *Reflection*	SESSION 4	Debate and Feedback	Communication of winning teams through peer evaluation and debate on DGD
*Continuous* *Evaluation and Improvement*	Implementation of Questionnaire Q3 (Q3.1 on technologies further promotes PC learning in the digital gender gap and Q3.2 on CT and DGD)		

**Table 3:** Topics covered in the activity on the Digital Gender Divide and Critical Thinking.

Group Activity		Topics
SP1 vs. SP2	A	The DGD and Internet advertising: Myth or reality?
SP3 vs. SP4	B	The DGD is it smaller in world powers (USA, China)?
INT1	A	The DGD and Internet advertising: myth or reality?
INT2	B	The DGD is it smaller in world powers (USA, China)?
INT3	C	Has the DGD reduced since COVID-19?

**Table 4:** Students’ lack of prior experience of the proposed activities to promote CT.

Activity Code	SP Group		INT Group	
	Women (5)	Men (13)	Women (5)	Men (9)
A1	80%	70%	NU ^1^	NU ^1^
A2	80%	70%	20%	45%
A3	100%	85%	80%	45%
A4	80%	92%	40%	89%
A5	20%	39%	40%	11%

^1^ NU = Not Undertaken: This activity was not carried out by students as it took place at the beginning of the course and many of them, due to the pandemic, had not yet joined the classes.

**Table 5:** Session 2 conclusions on Learning process with the materials provided to learn how to work with critical thinking.

Group	Response	F ^1^
SP1	“*Observations of coincidences: The results obtained have generally been the same and in the same order, in this case it has standardized the message regardless of whether we are male or female*”.	1st
SP3	*“[…] The girls have obtained the same links which, in turn, are different from those of boys;—The boys who play football have obtained the same links and a Nike advertisement; In the first search we all got the same results, but in the second search, when some used normal Google and others academic Google, we have different ones. […] [.] We have had a hard time finding reliable information, as we have had to compare the searches, since the first ones are not more important.”*	2 nd
INT3	*“Gathering the right information was not an easy step. Indeed, search engines provide us with many articles but choosing the most relevant ones is a crucial step that requires time. Concerning the personal criticism, we only asked ourselves about the digital divide at the beginning, after reading some articles, we could understand the subject. […]”*	3rd

^1^ F = Feedback on search N with keywords from a totally unknown topic.

**Table 6:** Reflections on the digital gender divide and critical thinking.

Teams/ Sources ^1^	ITCs & DGD		CT
SP1	*“* *Young people between the ages of 16 and 24 are currently dominating the Information Society”*	*“[…] it does not mean that there are no longer digital divides”*	*“[…] seen the studies and examples of the most used web pages, social networks, and search engines today”*
SP1	*“* *Children between the ages of 10 and 15 are becoming more and more manipulated by ICT”*	*“* *We had an idea that there was likely to be a gender gap.”*	
Sources SPI	Number of sources used: 4/Relevance: 4 and 5. e.g., Information society statistics. Eurostat 2020 (Population that has used the internet in the last 3 months per period. Spain, ue-27 and ue-28).		
SP2	*“* *Making visible the role of women in high positions in ICTs”*	*“* *The digital divide is real, but it has a solution”*	*“* *We have understood that there is a lot of work to be done. We have realized that by using critical thinking”*
SP2		*“[…] eliminate stereotypes, create social and collaborative awareness, inclusive language and less normalization of the “normal””*	
Sources SP2	Number of sources used: 6/Relevance 3 and 4. e.g., articles in national newspapers; https://elpais.com/elpais/2020/07/10/mujeres/1594372813_863855.html (accessed on 14 April 2021).		
SP3	*“* *Quality digital education is needed. Facilitating access to technology”*	*“[…] importance of bridging the digital gender divide […]”*	*“[…] it is more serious in underdeveloped economies, we must act for the future*”
SP3		*“* *Gender roles and stereotypes need to be eliminated”*	
Sources SP3	Number of sources used: 7/Relevance 3 and 4. e.g., reports; https://digitalpolicylaw.com/brecha-digital-de-genero-se-agrava-a-17-en-el-mundo-advierte-la-uit/ (accessed on 14 April 2021).		
SP4	*“* *Thinking that ICT is difficult”*	*“* *Yes, there is a gender gap”*	*“[…] this issue is present in all countries and it is important to reduce the gender gap”*
SP4	*“* *Stereotypes.; Lack of female role models in these professions”*	*“Women’s low preference for STEM careers”*	
Sources SP4	Number of sources used: 2/Relevance 4. e.g., U.S. digital financial and business news outlet, published by Insider https://www.businessinsider.com/the-countries-with-the-highest-and-lowest-gender-gap-around-the-world-2018-12 (accessed on 14 April 2021).		
INT1	*“The Internet is free to use”*	*“[…] gender digital divide and internet advertising is a myth or reality, is a myth but partially”*	*“The activity has encouraged to discussion and critical thinking”*
INT1		*“The gender gap in access to the Internet or information in developed and developing countries has disappeared”*	
INT1		*“The digital gender gap is becoming more and more of a myth”*	
Sources INT1	None		
INT2		*“[…] the first part of the hypothesis is true”*	*“[…] interesting to put critical thinking into practice”*
INT2		*“[…] (small gender gap in Europe). […]”*	*“we are able to make a critical judgment on the information offered. This is why this practice was interesting for us, in order to remind us to always check our sources”*
Sources INT2	Number of sources used: 7/Relevance 4 and 5. e.g., a platform that offers policy proposals to the G20. It is a new initiative of the Think 20 Engagement Group: “The G20 Insights Platform” https://www.g20-insights.org/policy_briefs/bridging-the-gender-digital-gap/ (accessed 15 April 2021); humanitarian organisation founded in 1937 that advances children’s rights and equality for girls. https://plan-international.org/education/bridging-the-digital-divide (accessed on 15 April 2021).		
INT3	*“[…] after such a big crisis, the difference between genders is so strong. “Giving girls and women access to digital resources, as well as the knowledge, […] will ensure that they are not further in an increasingly digital world”*	*“At first glance, we thought Covid would have had a positive impact on this digital gender divide.”*	*“Covid has brought many people to use technology (online courses, online shopping.) but also for calls for help, calls for information.”*
Sources INT3	Number of sources used: 5/Relevance 4 and 5. e.g., Report from a website that offers regular updates of FES regional projects and activities across Asia. Connected-Women-Gender-Gap.pdf (gsma.com) How COVID-19 fuels the digital gender divide: Friedrich-Ebert-Stiftung in Asia (fes.de)		

^1^ Sources: Number of sources used/Degree of relevance. Where 1 is “nothing relevant to the DGD” to 5 “very relevant”.

**Table 7:** Results of the AL (out of 10).

Group	Peer Evaluation Mark (0–10)				Teacher’s Mark (0–10)	
SP	SP1	8.2 vs.	SP2	7.7	8.7	9
	SP3	5.6 vs.	SP4	7.4	6.8	7.5
INT	INT1		7.3			7
	INT2		8.6			9
	INT3		7.8			8.5

**Table 8:** Descriptives of the activities proposed to promote CT. Average and Typical Error.

Activity Code	SP Group		INT Group	
	Women (5)	Men (13)	Women (5)	Men (9)
A1	4.20 (0.20)	4.23 (0.12)	NU ^1^	NU ^1^
A2	4.20 (0.37)	3.85 (0.27)	3.80 (0.58)	3.78 (0.40)
A3	4.40 (0.40)	3.37 (0.25)	3.40 (0.67)	3.78 (0.27)
A4	4.40 (0.24)	4.46 (0.18)	4.20 (0.37)	4.33 (0.23)
A5	3.60 (060)	3.77 (0.28)	3.60 (0.40)	3.11 (0.26)

^1^ NU = Not Undertaken: This activity was not carried out by INT students as it took place at the beginning of the course and many of them, due to the pandemic, had not yet joined the course.

**Table 9:** Descriptive of the skills acquired. Mean and Typical Error.

Skill Code	SP		INT	
	Women (9)	Men (11)	Women (5)	Men (9)
Skill 1	8.22 (0.32)	7.91 (0.34)	8.00 (0.70)	8.22 (0.40)
Skill 2	9.00 (0.44)	8.73 (0.30)	9.20 (0.37)	8.78 (0.54)
Skill 3	8.89 (0.45)	7.18 (0.35)	8.00 (0.54)	8.22 (0.46)

## Data Availability

The data presented in this study are available on request from the corresponding author. The data are not publicly available due to technical difficulties, owing to the diversity and size of the data used (e.g., recorded presentation sessions, submitted assignments, completed questionnaires, database, etc).
